# Hydroxygenkwanin Improves the Efficacy of Cytotoxic Drugs in ABCG2-Overexpressing Multidrug-Resistant Cancer Cells

**DOI:** 10.3390/ijms232112763

**Published:** 2022-10-23

**Authors:** Yan-Qing Li, Megumi Murakami, Yang-Hui Huang, Tai-Ho Hung, Shun-Ping Wang, Yu-Shan Wu, Suresh V. Ambudkar, Chung-Pu Wu

**Affiliations:** 1 Graduate Institute of Biomedical Sciences, College of Medicine, Chang Gung University, Taoyuan 33302, Taiwan; 2 Laboratory of Cell Biology, Center for Cancer Research, National Cancer Institute, NIH, Bethesda, MD 20892, USA; 3 Department of Physiology and Pharmacology, College of Medicine, Chang Gung University, Taoyuan 33302, Taiwan; 4 Department of Obstetrics and Gynecology, Taipei Chang Gung Memorial Hospital, Taipei 10507, Taiwan; 5 Department of Medicine, College of Medicine, Chang Gung University, Taoyuan 33302, Taiwan; 6 Department of Obstetrics and Gynecology, Keelung Chang Gung Memorial Hospital, Keelung 20401, Taiwan; 7 Department of Orthopedics, Taichung Veterans General Hospital, Taichung 40704, Taiwan; 8 Department of Chemistry, Tunghai University, Taichung 40704, Taiwan

**Keywords:** ABC-transporter, multidrug resistance, chemosensitizer, natural products, ABCG2, hydroxygenkwanin

## Abstract

Hydroxygenkwanin, a flavonoid isolated from the leaves of the *Daphne genkwa* plant, is known to have pharmacological properties; however, its modulatory effect on multidrug resistance, which is (MDR) mediated by ATP-binding cassette (ABC) drug transporters, has not been investigated. In this study, we examine the interaction between hydroxygenkwanin, ABCB1, and ABCG2, which are two of the most well-characterized ABC transporters known to contribute to clinical MDR in cancer patients. Hydroxygenkwanin is not an efflux substrate of either ABCB1 or ABCG2. We discovered that, in a concentration-dependent manner, hydroxygenkwanin significantly reverses ABCG2-mediated resistance to multiple cytotoxic anticancer drugs in ABCG2-overexpressing multidrug-resistant cancer cells. Although it inhibited the drug transport function of ABCG2, it had no significant effect on the protein expression of this transporter in cancer cells. Experimental data showing that hydroxygenkwanin stimulates the ATPase activity of ABCG2, and in silico docking analysis of hydroxygenkwanin binding to the inward-open conformation of human ABCG2, further indicate that hydroxygenkwanin sensitizes ABCG2-overexpressing cancer cells by binding to the substrate-binding pocket of ABCG2 and attenuating the transport function of ABCG2. This study demonstrates the potential use of hydroxygenkwanin as an effective inhibitor of ABCG2 in drug combination therapy trials for patients with tumors expressing higher levels of ABCG2.

## 1. Introduction

ATP-binding cassette (ABC) transporters are a superfamily of proteins that utilize the chemical energy derived from ATP hydrolysis to translocate compounds, including many of the most commonly prescribed anticancer drugs, across biological membranes [1,2]. Two of the most well-studied ABC transporters are ABC subfamily B member 1 (ABCB1; P-glycoprotein) and ABC subfamily G member 2 (ABCG2; BCRP), which are known to regulate the absorption of substrate drugs in the intestinal epithelium, and the tissue distribution of substrate drugs across the blood–brain barrier (BBB) and the blood–placenta barrier (BPB) [2,3,4,5]. Moreover, ABCB1 or ABCG2 overexpression in cancer cells is often associated with multidrug resistance (MDR) and poor prognosis in patients with solid tumors [6,7,8,9] or hematologic cancers [10,11,12,13,14,15,16]. Given that the development of MDR remains a major obstacle in cancer treatment [2,17], the use of a selective inhibitor against the activity of ABCB1 or ABCG2 has become an attractive therapeutic option to re-sensitize multidrug-resistant cancer cells to anticancer drugs [18]; however, due to the lack of selectivity or unexpected clinical side effects resulting from new combination therapies [2,19,20,21,22,23], there are currently no U.S. Food and Drug Administration (FDA)-approved agents for clinical application against multidrug-resistant cancers.

The potential application of inherently well-tolerated, plant-derived, bioactive compounds for the sensitization of multidrug-resistant cancer cells has been explored as an alternative approach [24,25]. Numerous natural products are known to be capable of reversing MDR in ABCB1- and ABCG2-overexpressing multidrug-resistant cancer cells [25,26,27,28,29]. Notably, many novel anticancer compounds derived from natural sources have shown promising results in preclinical and clinical trials [30,31,32]. Hydroxygenkwanin is a flavonoid isolated from leaves of the *Daphne genkwa* Sieb.et Zucc plant, which is commonly known as blue daphne or lilac daphne, and it is native to East Asia [33]. It has been found to exhibit anti-inflammatory and immunomodulatory properties [34,35], antimicrobial and antifungal activities [36], and it promotes pigmented hair regeneration [37]. In addition, the antiproliferative activity of hydroxygenkwanin has been demonstrated in a variety of cancer cell lines and animal models [38,39,40,41,42,43,44,45]; however, the potential chemosensitizing effect of hydroxygenkwanin on multidrug-resistant cancer cells has not been studied.

In this study, we investigated the potential reversal effect of hydroxygenkwanin on MDR, mediated by ABCB1 and ABCG2. We discovered that at low, sub-toxic concentrations, hydroxygenkwanin could selectively re-sensitize ABCG2-overexpressing multidrug-resistant cancer cells to cytotoxic anticancer drugs. Moreover, we demonstrated that hydroxygenkwanin reverses ABCG2-mediated MDR by modulating the drug efflux function of ABCG2, without altering its expression at the protein level in these cancer cells. These findings were further supported by data from ATPase assays and molecular docking analysis. Overall, our study demonstrates the potential of using hydroxygenkwanin as a chemotherapy adjuvant to improve the efficacy of anticancer drugs.

## 2. Results

### 2.1. Hydroxygenkwanin Sensitizes ABCG2-Overexpressing Multidrug-Resistant Cells to Cytotoxic Anticancer Drugs

**Figure 1:**
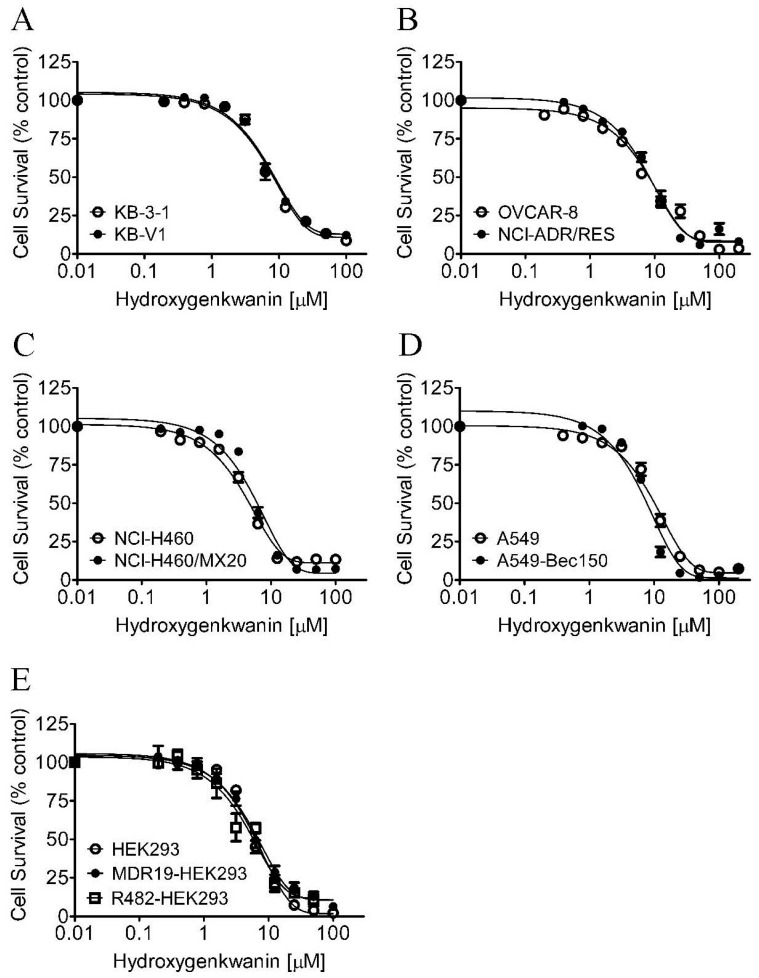
Intrinsic cytotoxicity of hydroxygenkwanin in normal and ABCB1- and ABCG2-overexpressing cell lines. The cytotoxicity of hydroxygenkwanin was determined in (**A**) the human epidermal cancer KB-3-1 cell line (empty circles) and its ABCB1-overexpressing subline KB-V1 (filled circles), (**B**) the human ovarian cancer OVCAR-8 cell line (empty circles) and its ABCB1-overexpressing subline NCI-ADR-RES (filled circles), (**C**) the human NSCLC NCI-H460 cell line (empty circles) and its ABCG2-overexpressing subline NCI-H460/MX20 (filled circles), (**D**) the human NSCLC A549 cell line (empty circles) and its ABCG2-overexpressing subline A549-Bec150 (filled circles), as well as in (**E**), the parental pcDNA3.1-HEK293 (empty circles), MDR19-HEK293 (HEK293 cells transfected with human ABCB1, filled circles), and R482-HEK293 (HEK293 cells transfected with human ABCG2, empty squares) cell lines. Points: mean values from at least three independent experiments; bars: SEM.

**Figure 2:**
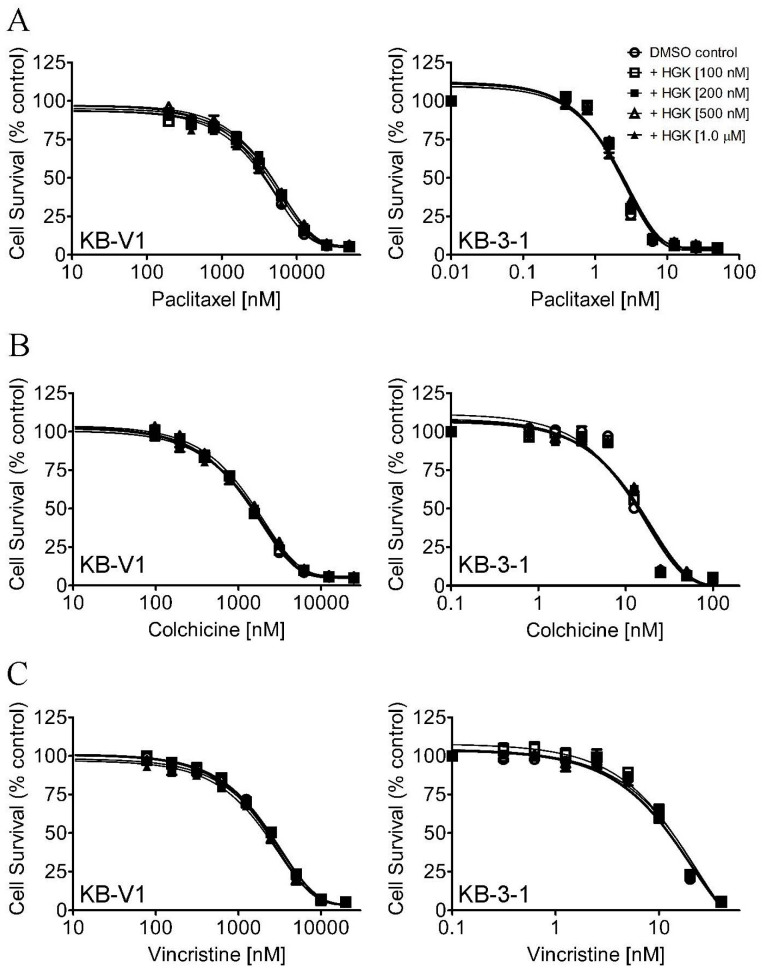

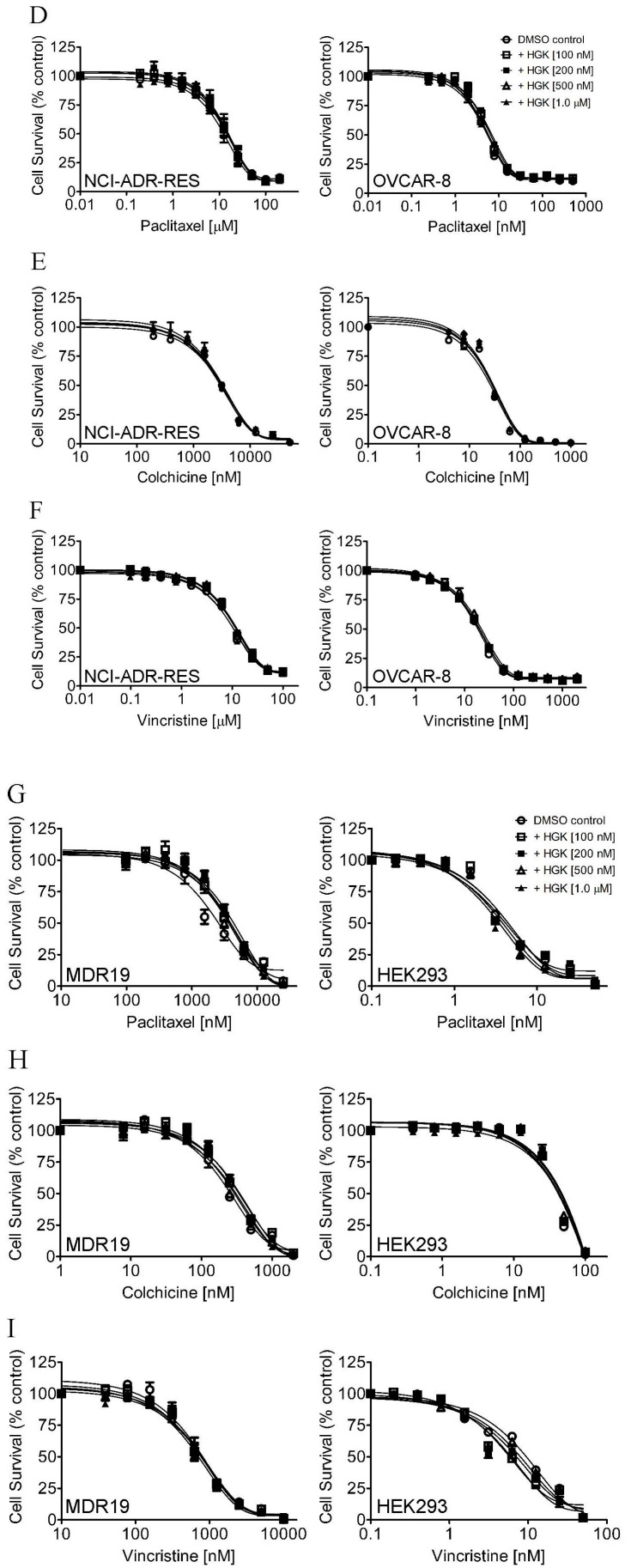
Cytotoxicity of ABCB1 substrate drugs in the absence or presence of hydroxygenkwanin. The cytotoxicity of paclitaxel, colchicine, and vincristine was determined in (**A**–**C**) KB-V1 and KB-3-1, (**D**–**F**) NCI-ADR-RES and OVCAR-8, and (**G**–**I**) MDR19-HEK293 and pcDNA3.1-HEK293 cells in the presence of DMSO (open circles) or hydroxygenkwanin (HGK) at 100 nM (open squares), 200 nM (filled squares), 500 nM (open triangles), or 1.0 μM (filled triangles), as described in the Section 4. Points, mean values from at least three independent experiments; bars; SEM.

**Figure 3:**
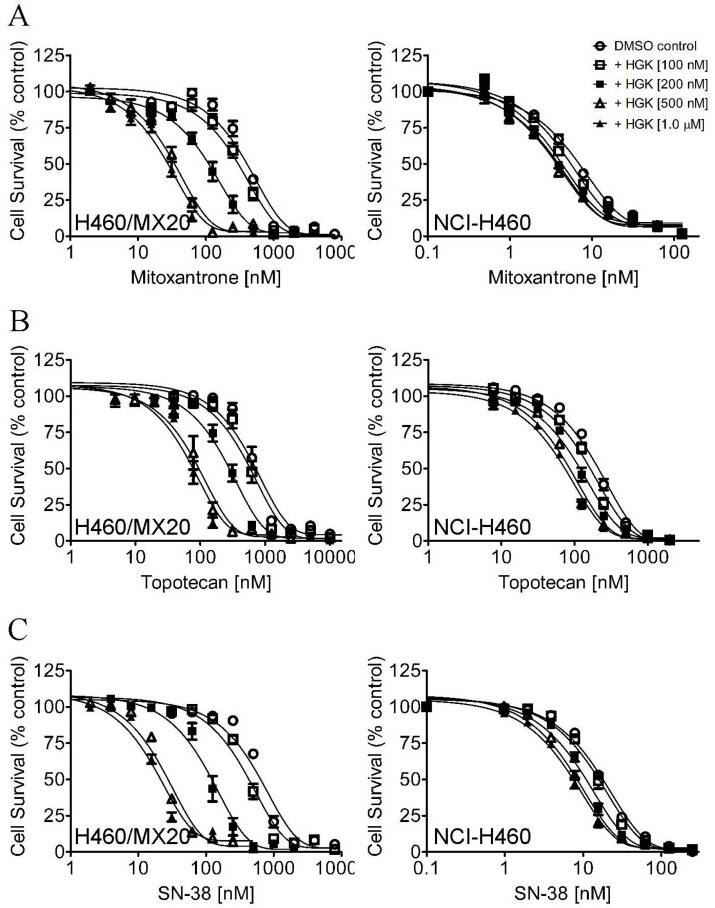

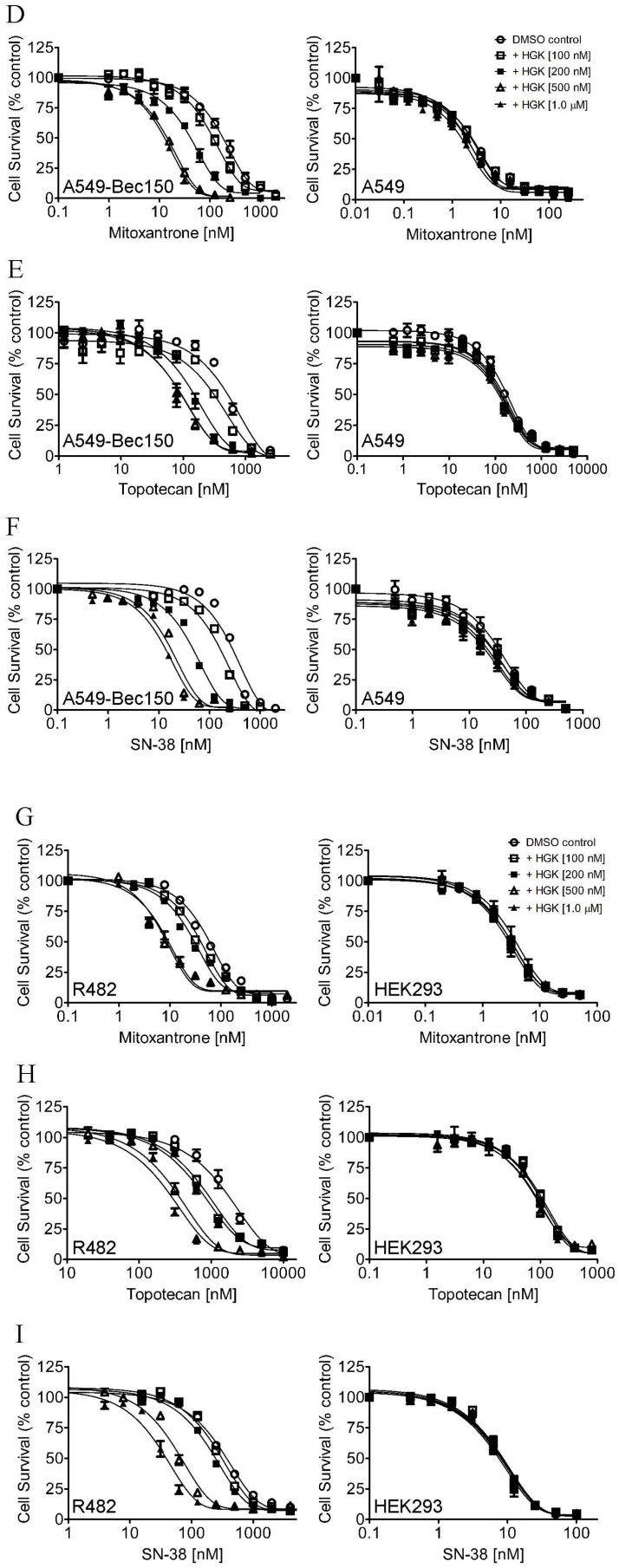
Cytotoxicity of ABCG2 substrate drugs in the absence or presence of hydroxygenkwanin. The cytotoxicity of mitoxantrone, topotecan, and SN-38 was determined in (**A**–**C**) NCI-H460/MX20 and NCI-H460, (**D**–**F**) A549-Bec150 and A549, and (**G**–**I**) R482-HEK293 and pcDNA3.1-HEK293 cells in the presence of DMSO (open circles) or hydroxygenkwanin (HGK) at 100 nM (open squares), 200 nM (filled squares), 500 nM (open triangles), or 1.0 μM (filled triangles) as described in the Section 4. Points, mean values from at least three independent experiments; bars; SEM.

First, the intrinsic cytotoxicity of hydroxygenkwanin was determined in the ABCB1-overexpressing cancer cell lines KB-V1 (Figure 1A) and NCI-ADR-RES (Figure 1B); in the ABCG2-overexpressing cancer cell lines NC-H460/MX20 (Figure 1C) and A549-Bec150 (Figure 1D); and in the respective drug-sensitive parental cancer cell lines KB-3-1, OVCAR-8, NCI-H460, and A549. Its cytotoxicity was also determined in pcDNA3.1-HEK293, ABCB1-transfected MDR19-HEK293, and ABCG2-transfected R482-HEK293 cell lines (Figure 1E). Based on cell survival curves (Figure 1), and the calculated IC_50_ values of hydroxygenkwanin (Table 1), the highest sub-toxic concentration of 1 μM hydroxygenkwanin was used to evaluate its chemosensitizing effect on MDR, and it was mediated by ABCB1 and ABCG2. The cytotoxicity of the ABCB1 substrates [46], paclitaxel, colchicine, and vincristine, was determined in KB-V1 and KB-3-1 (Figure 2A–C), NCI-ADR-RES and OVCAR-8 (Figure 2D–F), and MDR19-HEK293 and pcDNA3.1-HEK293 (Figure 2G–I) cell lines, whereas the cytotoxicity of the ABCG2 substrates [47,48,49], mitoxantrone, topotecan, and SN-38, was determined in NCI-H460/MX20 and NCI-H460 (Figure 3A–C), A549-Bec150 and A549 (Figure 3D–F), and R482-HEK293 and pcDNA3.1-HEK293 (Figure 3G–I) cell lines, in the absence or presence of hydroxygenkwanin. Our results show that hydroxygenkwanin had no significant effect on the cytotoxicity of ABCB1 substrate drugs in ABCB1-overexpressing cell lines (Table 2). In contrast, we found that hydroxygenkwanin significantly re-sensitized ABCG2-overexpressing cell lines to ABCG2 substrate drugs in a concentration-dependent manner (Table 3). Moreover, with the exception of the endogenous ABCG2-expressing NCI-H460 [50] and A549 [51] cell lines, hydroxygenkwanin (0.1–1.0 μM) had no significant effect on the cytotoxicity of substrate drugs in the parental cell lines. Of note, tariquidar and Ko143 were reference inhibitors; they were used as positive controls to fully re-sensitize ABCB1- and ABCG2-overexpressing multidrug-resistant cells to cytotoxic anticancer drugs. The fold-reversal (FR) value [52] was calculated by dividing the IC_50_ value of a known substrate drug by the IC_50_ value of the same substrate drug in the presence of hydroxygenkwanin or a reference inhibitor, thus signifying the extent of sensitization as a result of hydroxygenkwanin or a reference inhibitor in these multidrug-resistant cell lines.

### 2.2. Hydroxygenkwanin Attenuates the Drug Transport Function of ABCG2

**Figure 4:**
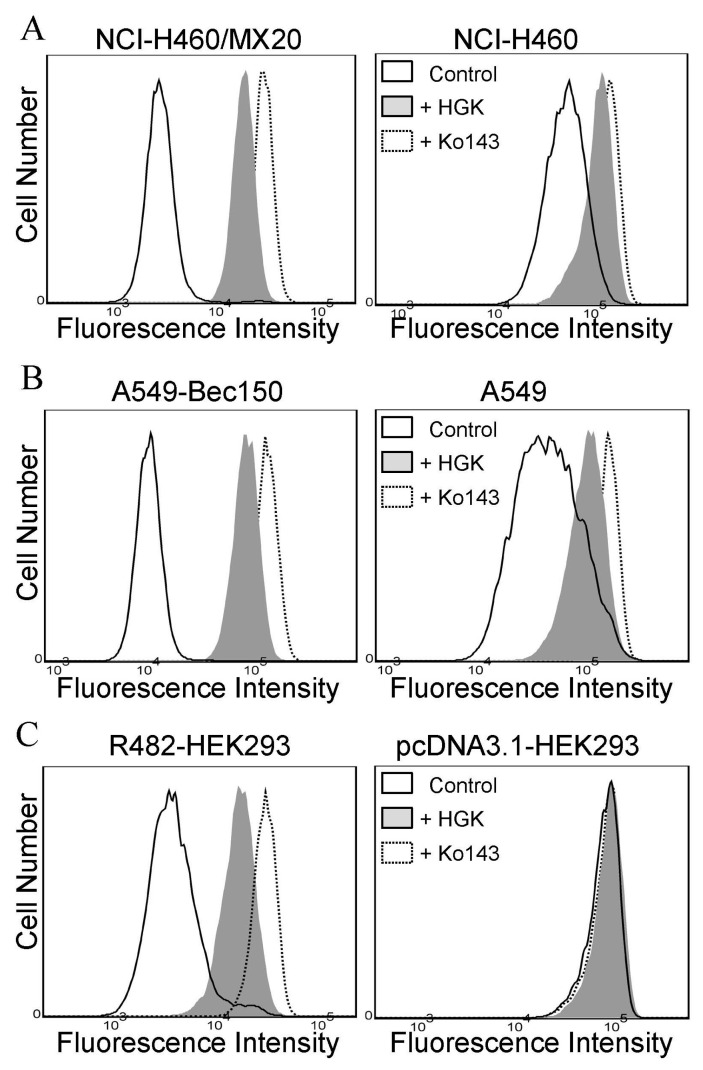
Hydroxygenkwanin attenuates ABCG2-mediated fluorescent substrate transport. The intracellular accumulation of a known fluorescent substrate, pheophorbide A (PhA), was determined in (**A**) NCI-H460 and NCI-H460/MX20 cancer cell lines, (**B**) A549 and A549-Bec150 cancer cell lines, and (**C**) pcDNA3.1-HEK293 and R482-HEK293 cell lines, in the presence of DMSO (control, solid line), 1 μM hydroxygenkwanin (+HGK, gray-shaded solid line), or 5 μM Ko143 (+Ko143, dotted line), which was used as a positive control for ABCG2. The fluorescent signals were analyzed with flow cytometry, as described in the Section 4. Histograms are representative results from at least three independent experiments.

**Figure 5:**
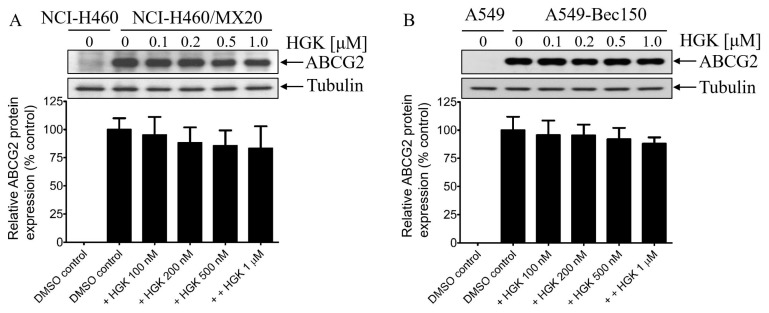
Hydroxygenkwanin does not alter the protein expression of ABCG2 in multidrug-resistant cancer cells. The ABCG2-overexpressing (**A**) NCI-H460/MX20 and (**B**) A549-Bec150 cancer cells were treated with DMSO (vehicle control) or hydroxygenkwanin (HGK) at 100 nM, 200 nM, 500 nM, or 1.0 μM for 72 h, and the cell lysates were processed for immunoblot detection and quantification, as described in the Section 4. Representative Western blots (top), and the corresponding quantification (bottom) of the human ABCG2 protein and α-tubulin as the internal loading control, are shown. Values are presented as the mean ± SD were obtained from at least three independent experiments.

Studies have reported that the direct inhibition [28,53,54,55,56,57], and/or transient down-regulation, of ABCG2 [58,59,60] are two of the most common mechanisms by which multidrug-resistant cancer cells become sensitized to cytotoxic anticancer drugs. To this end, we determined the effect of hydroxygenkwanin on the drug transport function and the protein expression of ABCG2 in ABCG2-overexpressing cells. To examine the effect of hydroxygenkwanin on ABCG2-mediated drug transport, ABCG2-overexpressing cells were incubated in IMDM that contained a known fluorescent substrate of ABCG2 PhA [61]. This occurred in the presence of DMSO (solid lines), 1 μM hydroxygenkwanin (filled solid lines), or 5 μM Ko143 (dotted lines), for a short period of 45 min, and they were processed as described in the Section 4. As expected, the intracellular accumulation of PhA in ABCG2-overexpressing multidrug-resistant cells (Figure 4, left panels) was considerably lower than in the corresponding parental cells (Figure 4, right panels). More importantly, we found that hydroxygenkwanin and Ko143 significantly restored the intracellular accumulation of PhA in ABCG2-overexpressing NCI-H460/MX20 (Figure 4A, left panel) and A549-Bec150 (Figure 4B, left panel) cancer cells, as well as in ABCG2-transfected R482-HEK293 cells (Figure 4C, left panel). Next, immunoblotting was performed to determine the protein expression of ABCG2 in ABCG2-overexpressing NCI-H460/MX20 and A549-Bec150 cancer cells treated with DMSO (control), or increasing concentrations of hydroxygenkwanin (0.1–1.0 μM), for 72 h, as detailed in the Section 4 (Figure S1). Our data show that hydroxygenkwanin did not significantly alter the protein expression of ABCG2 in NCI-H460/MX20 (Figure 5A) or A549-Bec150 (Figure 5B) cancer cells, thus suggesting that it restores the chemosensitivity of these multidrug-resistant cancer cells by attenuating the function of ABCG2.

### 2.3. Hydroxygenkwanin Stimulates the ATPase Activity of ABCG2

**Figure 6:**
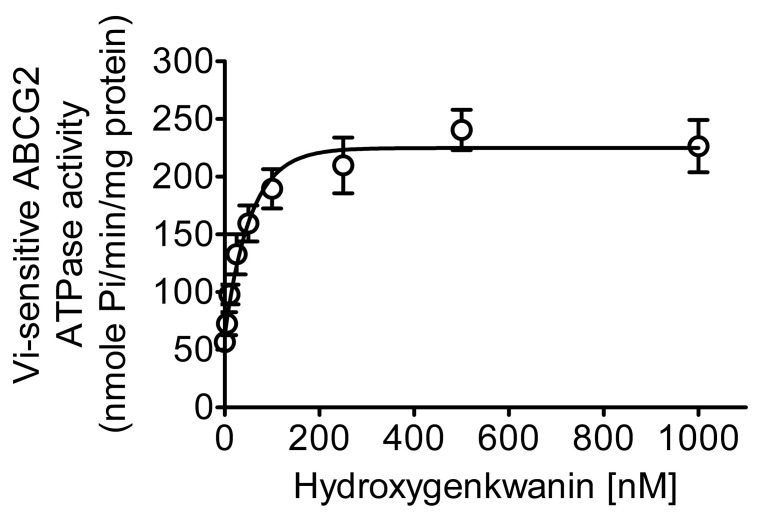
Hydroxygenkwanin stimulates ABCG2-mediated ATP hydrolysis. The effect of hydroxygenkwanin (0–1 μM) on the vanadate (Vi)-sensitive ATPase activity of ABCG2 was determined by endpoint P_i_ liberation assays using membrane vesicles prepared from ABCG2 baculovirus-infected High-Five insect cells, as previously described [65]. Points, mean from at least three independent experiments; bars, S.D.

Next, the effect of hydroxygenkwanin on the V_i_-sensitive ATPase activity of ABCG2 was determined to gain insight into the interaction between hydroxygenkwanin and ABCG2; this is because the substrates and inhibitors of ABCG2 are known to affect ABCG2-mediated ATP hydrolysis [62,63,64,65]. As shown in Figure 6, the Vi-sensitive ATPase activity of ABCG2 was stimulated by hydroxygenkwanin in a concentration-dependent manner, with a four-fold maximal stimulation (basal activity of 56.62 ± 7.08 nmole P_i_/min/mg protein) and a half-maximal effective concentration value (EC_50_) of approximately 19 nM. Our data indicate that hydroxygenkwanin interacts with, and has a high affinity with, ABCG2, which is in accordance with previous reports that have demonstrated other chemosensitizing agents binding to the drug-binding pocket of ABCG2 and stimulating ABCG2-mediated ATP hydrolysis [52,54,66].

### 2.4. Docking of Hydroxygenkwanin in the Drug-Binding Pocket of ABCG2

**Figure 7:**
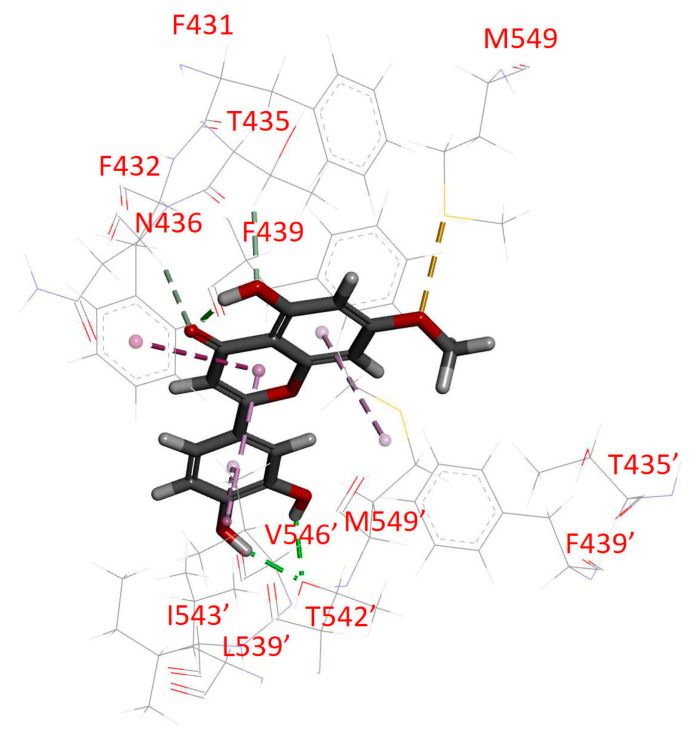
Docking of hydroxygenkwanin to ABCG2. The lowest energy binding mode of hydroxygenkwanin with the ABCG2 protein structure (PDB:6VXH) was predicted by using Accelrys Discovery Studio 4.0 software (Dassault Systemes BIOVIA, San Diego, CA, USA), as described in the Section 4. The stick representation (yellow) shows the molecular model of hydroxygenkwanin, and the predicted interacting amino acid atoms are represented by the following colors: carbon is colored gray; hydrogen is colored light gray; oxygen is colored red; and fluorine is colored cyan. Dotted lines represent the proposed interactions. The residues of monomer 2 are indicated by prime symbols.

We found that hydroxygenkwanin attenuates ABCG2-mediated drug efflux, as shown after an in silico molecular docking analysis of hydroxygenkwanin in the inward-open structure of human ABCG2 (PDB:6VXH) [67] was performed in order to gain insight into the key amino acid residues that interact with hydroxygenkwanin within the substrate-binding pocket of ABCG2. The lowest energy binding pose indicated numerous common interactions that were reported between substrates/inhibitors and ABCG2 [67,68,69]. The hydrophobic residues, Phe423, Val546′, and Met549′, were predicted to interact with the chromen-4-one core structure and phenyl substituent of hydroxygenkwanin via Pi–Pi/Pi–alkyl interactions (Figure 7). Hydrogen bonds were likely to form between the oxygen atoms on the ligand and Thr435, Asn436, and Thr542′ residues. Residue Met549 was shown to interact via S-X bonding. Thr435, Asn436, and Met549 were previously found to be particularly important residues affecting the function of the ABCG2 transporter. The interaction with Asn436 was found to be important for substrate binding [70], whereas the interaction with Thr435 was shown to be crucial for inhibition via inhibitor Ko143. A mutation of Met549 was reported to reduce drug transport [71].

## 3. Discussion

The ability of ABCG2 to transport a wide range of conventional anticancer drugs and molecularly targeted agents contributes to poor clinical outcomes in cancer patients [2,17]. Regrettably, the development of clinically applicable synthetic inhibitors of ABCG2 has been met with limited success, mostly due to the lack of selectivity [72], high toxicity [73], poor metabolic stability [74], and unexpected adverse reactions to them [2,57]; therefore, researchers have explored alternate approaches, such as the modulating effect of medicinal plant extracts that have been used in traditional Chinese medicine for hundreds of years. They have been studied as possible chemotherapy adjuvants for cancer treatment [25,26,27]. Moreover, natural compounds such as flavonoids [75,76], terpenoids [77], and chalcones [28,78] were found to modulate the function of ABCG2, whereas genistein [79], curcumin, and epigallocatechin gallate (EGCG) [80] were found to modulate the expression of ABCG2 in cancer cell lines. Hydroxygenkwanin is a flavonoid isolated from *Daphne genkwa* Sieb.et Zucc, a well-known traditional Chinese medicinal plant [33]. Previous studies reported that hydroxygenkwanin could suppress the progression of non-small cell lung cancer (NSCLC) [41], oral squamous cell carcinoma (OSCC) [43], and glioma cells [45]. Moreover, hydroxygenkwanin was shown to suppress the cell growth and invasion of hepatocellular carcinoma cells (HCC) by inducing the expression of the microRNA, miR-320a [42]. Recently, hydroxygenkwanin was found to enhance the chemosensitivity of HCC cells by inhibiting the expression of class I histone deacetylase (HDAC) [40] and by inhibiting the DNA damage response [39]. In this study, we investigated the modulatory effect of hydroxygenkwanin on ABCB1- and ABCG2-mediated MDR in multidrug-resistant cancer cells.

**Figure 8:**
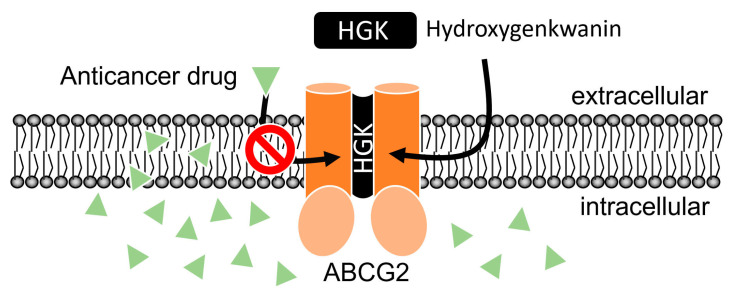
A schematic illustration of hydroxygenkwanin (HGK) reversing ABCG2-mediated drug resistance in cancer cells by inhibiting the drug transport function of ABCG2. HGK binds to the drug-binding pocket of ABCG2 and competes with the binding of substrate anticancer drugs of ABCG2 (green triangles), resulting in elevated intracellular concentration and the cytotoxicity of anticancer drugs in ABCG2-overexpressing multidrug-resistant cancer cells.

First, the intrinsic cytotoxicity of hydroxygenkwanin was determined in drug-sensitive parental cell lines and multidrug-resistant sublines overexpressing ABCB1 or ABCG2. We found that hydroxygenkwanin is equally cytotoxic to both parental and multidrug-resistant cell lines, thus suggesting that it is not rapidly transported out of cancer cells by either ABCB1 or ABCG2 (Figure 1). More importantly, we discovered that at sub-toxic concentrations, hydroxygenkwanin enhanced the cytotoxicity of the ABCG2 substrates, mitoxantrone, SN-38, and topotecan, in ABCG2-overexpressing cells (Figure 3), but it had no significant effect on the cytotoxicity of the ABCB1 substrates, colchicine, vincristine, and paclitaxel in ABCB1-overexpressing cells (Figure 2). Our results demonstrate that hydroxygenkwanin is a more potent inhibitor of ABCG2, compared with ABCB1, and is more specific to ABCG2. It is worth noting that in previous studies, high concentrations of hydroxygenkwanin (20–50 μM) were used in biochemical assays [39,40,41,42]. In contrast, at the highest tested concentration of 1 μM, hydroxygenkwanin reversed ABCG2-mediated MDR in a manner similar to the reference inhibitor, Ko143 (Table 3). To explore the potential mechanism of hydroxygenkwanin sensitizing ABCG2-overexpressing cells, the effect of hydroxygenkwanin on the drug transport function and protein expression of ABCG2 was examined in ABCG2-overexpressing cells. We found that ABCG2-mediated drug efflux in ABCG2-overexpressing cancer cells and ABCG2-transfected HEK293 cells was strongly inhibited by hydroxygenkwanin (Figure 4). On the other hand, the total protein level of ABCG2 in ABCG2-overexpressing cancer cells lines was not significantly altered as a result of incubation with hydroxygenkwanin for 72 h (Figure 5). The molecular docking simulation of hydroxygenkwanin binding to ABCG2 predicted several interactions, with residues of TM2 and TM5, from both monomers, in the substrate binding cavity (Figure 7). The interacting residues showed a high resemblance to the residues contributing to the binding of mitoxantrone to ABCG2 proteins in a cryo-EM study by Kowal et. al. [68]; therefore, molecular docking studies are in accordance with our results, as they also show the hydroxygenkwanin stimulation of the ATPase activity in ABCG2 in a concentration-dependent manner (Figure 6). Our data indicate that hydroxygenkwanin selectively reverses ABCG2-mediated MDR by competing with the binding of other substrate drugs in the substrate-binding pocket of ABCG2, hence inhibiting the drug transport activity of ABCG2 (Figure 8).

In summary, this study demonstrates that hydroxygenkwanin is a potent and selective modulator for ABCG2. Collectively, our findings indicate that further study is needed to evaluate its potential use as a chemotherapy adjuvant to improve the efficacy of anticancer drugs in patients with tumors expressing relatively high levels of ABCG2.

## 4. Materials and Methods

### 4.1. Chemicals

Hydroxygenkwanin was purchased from Selleckchem (Houston, TX, USA). The TOOLS Cell Counting (CCK-8) kit was acquired from Biotools Co., Ltd. (Taipei, Taiwan). Paclitaxel, vincristine, colchicine, mitoxantrone, SN-38, topotecan, and all other chemicals were obtained from Sigma (St. Louis, MO, USA), unless otherwise stated.

### 4.2. Cell Lines

The ABCB1-overexpressing KB-V1 cell line, derived from human epidermal cancer, was maintained in DMEM supplemented with 1 μg/mL of vinblastine [81]. The ABCB1-overexpressing NCI-ADR-RES cell line (human ovarian cancer) was maintained in RPMI 1640 supplemented with 0.85 μM doxorubicin [82]. The ABCG2-overexpressing NCI-H460/MX20 cell line (human non-small cell lung cancer (NSCLC)) was maintained in RPMI 1640 supplemented with 20 nM mitoxantrone [83]. The ABCG2-overexpressing A549-Bec150 cell line (human NSCLC) was maintained in RPMI 1640 supplemented with 150 nM becatecarin [84]. Parental KB-3-1, OVCAR-8, NCI-H460, and A549 cells were cultured in media, without vinblastine, doxorubicin, mitoxantrone, or becatecarin. Parental human embryonic kidney (HEK293) cells, transfected with the empty pcDNA 3.1 vector, human ABCB1 or human ABCG2 (referred to as pcDNA3.1-HEK293, MDR19-HEK293, and R482-HEK293, respectively), were maintained in DMEM supplemented with 2 mg/mL of G418, as previously noted [85,86]. All cell lines were maintained at 37 °C, in 5% CO_2_ humidified air, and grown in media supplemented with 10% fetal calf serum (FCS), 2 mM _L_-glutamine, and 100 units/mL of a penicillin–streptomycin solution mixture; then, the cell lines were screened periodically for mycoplasma contamination using a TOOLS Mycoplasma Detection Kit. The cell lines were kindly provided by Drs. Michael Gottesman and Susan Bates (NCI, NIH, Bethesda, MD, USA).

### 4.3. Cell Viability Assay

The cytotoxicity of cytotoxic drugs was determined by standard MTT and CCK-8 assays, as previously noted [87,88]. Briefly, cells were plated in 96-well flat-bottom plates and allowed to attach overnight at 37 °C, in 5% CO_2_ humidified air. Cytotoxic drugs were subsequently added at various concentrations, in the absence or presence of hydroxygenkwanin or a reference inhibitor (0.5% (*v*/*v*) final concentration of DMSO in all wells), for an additional 72 h before being processed, as previously noted [66]. The 50% inhibitory concentration (IC_50_) value of each drug regimen was calculated using a fitted concentration–response curve obtained from at least three independent experiments.

### 4.4. Flow Cytometry

Flow cytometry assays, with the ABCG2 substrate, pheophorbide A (PhA) (395 nm excitation and 670 nm emission), were performed, as previously noted [61,89]. Briefly, trypsinized cells were incubated in Iscove’s modified Dulbecco’s medium (IMDM) containing 10% FCS with 1 µM PhA in the presence of DMSO (control), 1 μM hydroxygenkwanin, or 5 μM Ko143, a known inhibitor of ABCG2. The relative fluorescence intensity of PhA was measured using a FACScan Flow Cytometer (BD Biosciences San Jose, CA, USA), and analyzed using CellQuest software (BD Biosciences, CA, USA) and FlowJo software (Tree Star, Inc., Ashland, OR, USA), as previously noted [64].

### 4.5. Immunoblotting

An immunoblot assay for the detection of human ABCG2, with the positive loading control, tubulin, was performed using BXP-21 (1:1000 dilution) and anti-alpha tubulin (1:100,000 dilution) (Sigma-Aldrich, St. Louis, MO, USA) antibodies, respectively, as previously noted [66].

### 4.6. ATPase Assay

The vanadate (Vi)-sensitive ATPase activity of ABCG2 was determined based on the endpoint P_i_ assay, using total membrane vesicles prepared from ABCG2 baculovirus-infected High-Five insect cells, as previously noted [65,90].

### 4.7. Docking Analysis

The ABCG2 protein (PDB:6VXH) [67] and ligand preparation were performed using the CDOCKER module of Accelrys Discovery Studio 4.0. The CHARMM force field was used for energy minimization for the protein and hydroxygenkwanin structures. The conformation of the ligand with the lowest CDOCKER interaction energy was used for docking analysis, as previously noted [91].

### 4.8. Data Analysis

Experimental values are presented as an average ± standard deviation (SD) or as an average ± standard error of the mean (SEM); they were calculated from dose–response data, from at least three independent experiments, using GraphPad Prism software (GraphPad Software, La Jolla, CA, USA) and KaleidaGraph software (Synergy Software, Reading, PA, USA). Unpaired two-tailed Student’s t-tests were performed to analyze the difference between experimental data and control, or improvement in fit data.

## Figures and Tables

**Table 1:** The cytotoxicity of hydroxygenkwanin in human cell lines overexpressing ABCB1 or ABCG2.

Cell Line	Transporter Expressed	IC_50_ (μM) ^1^	RF ^2^
KB-3-1	-	7.54 ± 1.25	1.0
KB-V1	ABCB1	7.94 ± 1.15	1.1
OVCAR-8	-	7.73 ± 0.64	1.0
NCI-ADR-RES	ABCB1	7.31 ± 1.34	0.9
NCI-H460	-	4.36 ± 0.83	1.0
NCI-H460/MX20	ABCG2	5.20 ± 1.32	1.2
A549	-	8.96 ± 1.80	1.0
A549-Bec150	ABCG2	5.93 ± 1.95	0.7
pcDNA3.1-HEK293	-	5.15 ± 1.20	1.0
MDR19-HEK293	ABCB1	6.10 ± 0.67	1.2
R482-HEK293	ABCG2	5.07 ± 0.86	1.0

Abbreviation: RF, resistance factor. ^1^ IC_50_ values were calculated from at least three independent experiments as described in the Section 4. ^2^ RF values were obtained by dividing the IC_50_ value of hydroxygenkwanin in the ABCB1- or ABCG2-overexpressing multidrug-resistant cell line by the IC_50_ value of hydroxygenkwanin in the corresponding drug-sensitive parental cell line.

**Table 2:** The effect of hydroxygenkwanin on reversing ABCB1-mediated multidrug resistance in drug-resistant human cell lines.

		Mean IC_50_^1^ ± SD and (FR ^2^)	
Treatment	Concentration (μM)	OVCAR-8 (Parental) [nM]	NCI-ADR-RES (Resistant) [μM]
Colchicine	-	22.31 ± 6.77 (1.0)	2.50 ± 0.51 (1.0)
+HGK	0.1	23.11 ± 6.57 (1.0)	2.70 ± 0.59 (0.9)
+HGK	0.2	22.74 ± 7.17 (1.0)	2.75 ± 0.58 (0.9)
+HGK	0.5	23.25 ± 7.58 (1.0)	2.71 ± 0.54 (0.9)
+HGK	1.0	20.92 ± 6.17 (1.1)	2.67 ± 0.51 (0.9)
+Tariquidar	1.0	22.67 ± 8.45 (1.0)	28.69 ± 8.59 [nM] ** (87)
		[nM]	[μM]
Vincristine	-	16.47 ± 2.36 (1.0)	10.25 ± 0.95 (1.0)
+HGK	0.1	17.97 ± 2.08 (0.9)	11.09 ± 1.18 (0.9)
+HGK	0.2	18.28 ± 2.18 (0.9)	11.75 ± 1.15 (0.9)
+HGK	0.5	20.25 ± 2.91 (0.8)	11.41 ± 1.33 (0.9)
+HGK	1.0	18.44 ± 2.21 (0.9)	10.83 ± 1.37 (0.9)
+Tariquidar	1.0	12.11 ± 1.93 (1.4)	95.48 ± 13.84 [nM] *** (107)
		[nM]	[μM]
Paclitaxel	-	5.11 ± 1.01 (1.0)	13.84 ± 1.29 (1.0)
+HGK	0.1	5.98 ± 1.21 (0.9)	13.03 ± 1.46 (1.1)
+HGK	0.2	6.69 ± 1.33 (0.8)	13.71 ± 1.58 (1.0)
+HGK	0.5	5.45 ± 1.06 (0.9)	13.42 ± 1.77 (1.0)
+HGK	1.0	5.31 ± 0.96 (1.0)	12.05 ± 1.22 (1.1)
+Tariquidar	1.0	4.09 ± 0.86 (1.2)	10.61 ± 2.21 [nM] *** (1304)
**Treatment**	**Concentration** **(μM)**	**KB-3-1 (Parental)** **[nM]**	**KB-V1 (Resistant)** **[μM]**
Colchicine	-	10.69 ± 4.21 (1.0)	1.19 ± 0.17 (1.0)
+HGK	0.1	11.03 ± 4.36 (1.0)	1.20 ± 0.16 (1.0)
+HGK	0.2	11.58 ± 4.53 (0.9)	1.28 ± 0.16 (0.9)
+HGK	0.5	11.89 ± 4.43 (0.9)	1.32 ± 0.17 (0.9)
+HGK	1.0	12.28 ± 4.92 (0.9)	1.23 ± 0.12 (1.0)
+Tariquidar	1.0	10.89 ± 4.32 (1.0)	10.29 ± 3.65 [nM] *** (115)
		[nM]	[μM]
Vincristine	-	10.03 ± 2.92 (1.0)	2.02 ± 0.33 (1.0)
+HGK	0.1	10.53 ± 3.09 (1.0)	2.04 ± 0.31 (1.0)
+HGK	0.2	10.95 ± 3.29 (0.9)	2.00 ± 0.31 (1.0)
+HGK	0.5	10.20 ± 2.87 (1.0)	1.89 ± 0.31 (1.1)
+HGK	1.0	10.18 ± 2.86 (1.0)	1.86 ± 0.30 (1.1)
+Tariquidar	1.0	10.33 ± 3.09 (1.0)	14.61 ± 3.87 [nM] *** (138)
		[nM]	[μM]
Paclitaxel	-	1.60 ± 0.55 (1.0)	3.41 ± 0.46 (1.0)
+HGK	0.1	1.67 ± 0.55 (1.0)	3.97 ± 0.58 (0.9)
+HGK	0.2	1.72 ± 0.50 (0.9)	4.17 ± 0.59 (0.8)
+HGK	0.5	1.81 ± 0.54 (0.9)	4.16 ± 0.51 (0.8)
+HGK	1.0	1.71 ± 0.46 (0.9)	3.43 ± 0.43 (1.0)
+Tariquidar	1.0	1.52 ± 0.46 (1.1)	1.50 ± 0.40 [nM] *** (2273)
**Treatment**	**Concentration** **(μM)**	**pcDNA3.1-HEK293 (Parental)** **[nM]**	**MDR19-HEK293 ** **(Resistant)** **[nM]**
Colchicine	-	33.96 ± 13.18 (1.0)	206.78 ± 40.87 (1.0)
+HGK	0.1	34.85 ± 12.59 (1.0)	269.33 ± 57.01 (0.8)
+HGK	0.2	36.92 ± 13.78 (0.9)	279.90 ± 67.09 (0.7)
+HGK	0.5	38.28 ± 13.96 (0.9)	236.33 ± 57.01 (0.9)
+HGK	1.0	35.99 ± 13.07 (0.9)	252.20 ± 60.70 (0.8)
+Tariquidar	1.0	30.91 ± 12.38 (1.1)	16.81 ± 5.14 ** (12.3)
		[nM]	[nM]
Vincristine	-	7.91 ± 1.18 (1.0)	635.22 ± 146.25 (1.0)
+HGK	0.1	7.88 ± 1.00 (1.0)	615.13 ± 118.64 (1.0)
+HGK	0.2	7.57 ± 1.04 (1.0)	659.88 ± 138.77 (1.0)
+HGK	0.5	9.41 ± 1.34 (0.8)	575.61 ± 117.02 (1.1)
+HGK	1.0	9.07 ± 1.21 (0.9)	678.52 ± 139.09 (0.9)
+Tariquidar	1.0	1.61 ± 0.32 *** (4.9)	1.92 ± 0.53 ** (330.8)
		[nM]	[μM]
Paclitaxel	-	3.61 ± 0.74 (1.0)	2.27 ± 0.35 (1.0)
+HGK	0.1	3.66 ± 0.82 (1.0)	3.16 ± 0.64 (0.7)
+HGK	0.2	3.70 ± 0.60 (1.0)	3.50 ± 0.84 (0.6)
+HGK	0.5	3.14 ± 0.74 (1.1)	2.96 ± 0.71 (0.8)
+HGK	1.0	2.77 ± 0.67 (1.3)	3.15 ± 0.76 (0.7)
+Tariquidar	1.0	2.79 ± 0.63 (1.3)	2.43 ± 0.52 [nM] *** (934.2)

Abbreviation: HGK, hydroxygenkwanin; FR, fold-reversal. ^1^ IC_50_ values are mean ± SD calculated from at least three independent experiments. ^2^ FR values were calculated by dividing the IC_50_ value of a known ABCB1 substrate drug by the IC_50_ value of the same substrate drug in the presence of HGK or tariquidar. ** *p* < 0.01; *** *p* < 0.001.

**Table 3:** The effect of hydroxygenkwanin on reversing ABCG2-mediated multidrug resistance in drug-resistant human cell lines.

		Mean IC_50_^1^ ± SD and (FR ^2^)	
**Treatment**	**Concentration** **(μM)**	**NCI-H460 (Parental)** **[nM]**	**NCI-H460/MX20 (Resistant)** **[nM]**
Mitoxantrone	-	5.96 ± 0.57 (1.0)	347.79 ± 100.33 (1.0)
+HGK	0.1	4.51 ± 0.66 * (1.3)	258.23 ± 66.78 (1.3)
+HGK	0.2	3.65 ± 0.54 ** (1.6)	94.65 ± 18.38 * (3.7)
+HGK	0.5	3.18 ± 0.39 ** (1.9)	27.57 ± 6.23 ** (12.6)
+HGK	1.0	3.28 ± 0.38 ** (1.8)	22.29 ± 5.14 ** (15.6)
+Ko143	1.0	3.07 ± 0.31 ** (1.9)	16.45 ± 4.82 ** (21.1)
		[nM]	[nM]
SN-38	-	14.09 ± 3.03 (1.0)	542.54 ± 165.97 (1.0)
+HGK	0.1	12.42 ± 2.84 (1.1)	349.80 ± 96.43 (1.6)
+HGK	0.2	8.47 ± 1.97 (1.7)	96.98 ± 23.89 * (5.6)
+HGK	0.5	6.39 ± 1.43 * (2.2)	21.78 ± 5.93 ** (24.9)
+HGK	1.0	5.82 ± 1.14 * (2.4)	18.00 ± 4.67 ** (30.1)
+Ko143	1.0	5.67 ± 1.14 * (2.5)	7.89 ± 2.17 ** (68.8)
		[nM]	[nM]
Topotecan	-	161.17 ± 39.96 (1.0)	588.67 ± 171.97 (1.0)
+HGK	0.1	126.09 ± 29.51 (1.3)	474.29 ± 148.18 (1.2)
+HGK	0.2	94.37 ± 20.54 (1.7)	224.01 ± 55.50 * (2.6)
+HGK	0.5	67.71 ± 18.07 * (2.4)	78.52 ± 20.25 ** (7.5)
+HGK	1.0	59.01 ± 12.75 * (2.7)	64.20 ± 18.15 ** (9.2)
+Ko143	1.0	73.03 ± 16.63 * (2.2)	63.52 ± 20.13 ** (9.3)
**Treatment**	**Concentration** **(μM)**	**A549** **(Parental)** **[nM]**	**A549-Bec150** **(Resistant)** **[nM]**
Mitoxantrone	-	3.06 ± 0.37 (1.0)	157.39 ± 14.89 (1.0)
+HGK	0.1	2.87 ± 0.35 (1.1)	99.73 ± 9.68 ** (1.6)
+HGK	0.2	2.11 ± 0.23 * (1.5)	36.36 ± 3.92 *** (4.3)
+HGK	0.5	2.12 ± 0.22 * (1.4)	11.98 ± 2.02 *** (13.1)
+HGK	1.0	1.79 ± 0.23 ** (1.7)	10.68 ± 2.27 *** (14.7)
+Ko143	1.0	1.91 ± 0.22 ** (1.6)	8.28 ± 1.37 *** (19.0)
		[nM]	[nM]
SN-38	-	30.31 ± 2.48 (1.0)	240.94 ± 81.57 (1.0)
+HGK	0.1	26.76 ± 3.12 (1.1)	135.77 ± 39.77 (1.8)
+HGK	0.2	25.78 ± 2.44 (1.2)	39.58 ± 9.87 * (6.1)
+HGK	0.5	22.33 ± 2.79 * (1.4)	15.55 ± 4.52 ** (15.5)
+HGK	1.0	21.14 ± 3.26 * (1.4)	12.25 ± 3.16 ** (19.7)
+Ko143	1.0	16.82 ± 2.12 ** (1.8)	8.35 ± 2.14 ** (28.9)
		[nM]	[nM]
Topotecan	-	160.60 ± 14.29 (1.0)	485.08 ± 127.55 (1.0)
+HGK	0.1	145.07 ± 20.61 (1.1)	301.71 ± 63.88 (1.6)
+HGK	0.2	157.89 ± 24.41 (1.0)	129.65 ± 26.63 ** (3.7)
+HGK	0.5	144.03 ± 21.82 (1.1)	82.21 ± 14.91 ** (5.9)
+HGK	1.0	132.91 ± 21.73 (1.2)	80.20 ± 14.59 ** (6.0)
+Ko143	1.0	110.15 ± 9.67 ** (1.5)	112.61 ± 15.05 ** (4.3)
**Treatment**	**Concentration** **(μM)**	**pcDNA3.1-HEK293 (Parental)** **[nM]**	**R482-HEK293** **(Resistant)** **[nM]**
Mitoxantrone	-	2.45 ± 0.34 (1.0)	50.95 ± 4.11 (1.0)
+HGK	0.1	2.78 ± 0.29 (0.9)	33.32 ± 2.99 ** (1.5)
+HGK	0.2	2.83 ± 0.45 (0.9)	26.47 ± 2.36 *** (1.9)
+HGK	0.5	2.71 ± 0.35 (0.9)	7.58 ± 1.17 *** (6.7)
+HGK	1.0	3.21 ± 0.42 (0.8)	8.49 ± 1.24 *** (6.0)
+Ko143	1.0	2.31 ± 0.31 (0.9)	6.02 ± 0.69 *** (8.5)
		[nM]	[nM]
SN-38	-	6.77 ± 1.46 (1.0)	321.48 ± 37.56 (1.0)
+HGK	0.1	6.86 ± 1.57 (1.0)	259.31 ± 48.57 (1.2)
+HGK	0.2	6.72 ± 1.41 (1.0)	205.59 ± 35.98 * (1.6)
+HGK	0.5	6.55 ± 1.44 (1.0)	57.54 ± 12.49 *** (5.6)
+HGK	1.0	6.12 ± 1.32 (1.1)	34.31 ± 7.71 *** (9.4)
+Ko143	1.0	5.83 ± 1.27 (1.2)	15.15 ± 3.62 *** (21.2)
		[nM]	[nM]
Topotecan	-	87.00 ± 18.29 (1.0)	1549.67 ± 314.57 (1.0)
+HGK	0.1	93.41 ± 17.88 (0.9)	811.39 ± 165.76 * (1.9)
+HGK	0.2	86.31 ± 15.14 (1.0)	717.27 ± 135.32 * (2.2)
+HGK	0.5	78.40 ± 13.49 (1.1)	307.01 ± 78.05 ** (5.0)
+HGK	1.0	79.30 ± 15.61 (1.1)	252.88 ± 61.04 ** (6.1)
+Ko143	1.0	88.95 ± 19.19 (1.0)	239.45 ± 60.24 ** (6.5)

Abbreviation: HGK, hydroxygenkwanin; FR, fold-reversal. ^1^ IC_50_ values are mean ± SD calculated from at least three independent experiments. ^2^ FR values were calculated by dividing the IC_50_ value of a known ABCG2 substrate drug by the IC_50_ value of the same substrate drug in the presence of HGK or Ko143. * *p* < 0.05; ** *p* < 0.01; *** *p* < 0.001.

## Data Availability

The data presented in this study are available on request from the corresponding author.

## Supplementary Materials

The following supporting information can be downloaded at: https://www.mdpi.com/article/10.3390/ijms232112763/s1, Figure S1: The effect of hydroxygenkwanin on the protein expression of ABCG2 in human NCI-H460 and NCI-H460/MX20 NSCLC cells, and human A549 and A549-Bec150 NSCLC cells.
