# Supplementary material for: Hydroxygenkwanin Improves the Efficacy of Cytotoxic Drugs in ABCG2-Overexpressing Multidrug-Resistant Cancer Cells
Source: Int J Mol Sci. 2022 Oct 23;23(21):12763. doi: 10.3390/ijms232112763 (PMC9658017; doi:10.3390/ijms232112763)
Supplement: Supplementary file 1 [file ijms-23-12763-s001.zip › ijms-1978228-supplementary.pdf]

Figure S1: The effect of hydroxygenkwanin on the protein expression of ABCG2 in human NCI-H460 and NCI-H460/MX20 NSCLC cells, and human A549 and A549-Bec150 NSCLC cells

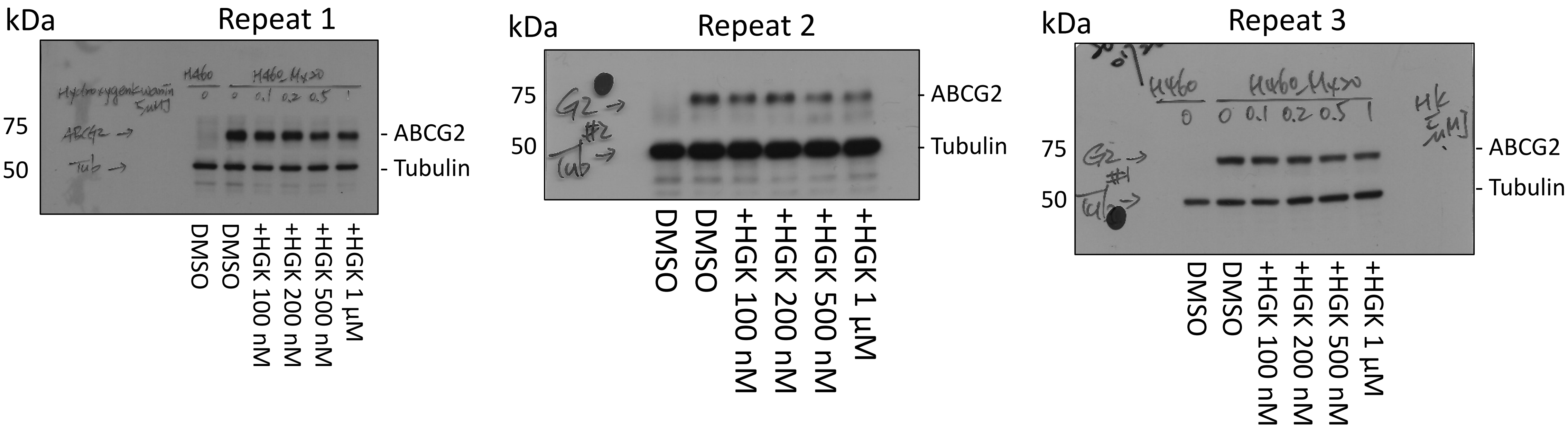

Original western blots for Figure 5A (NCI-H460/MX20)

Figure S1: Continued

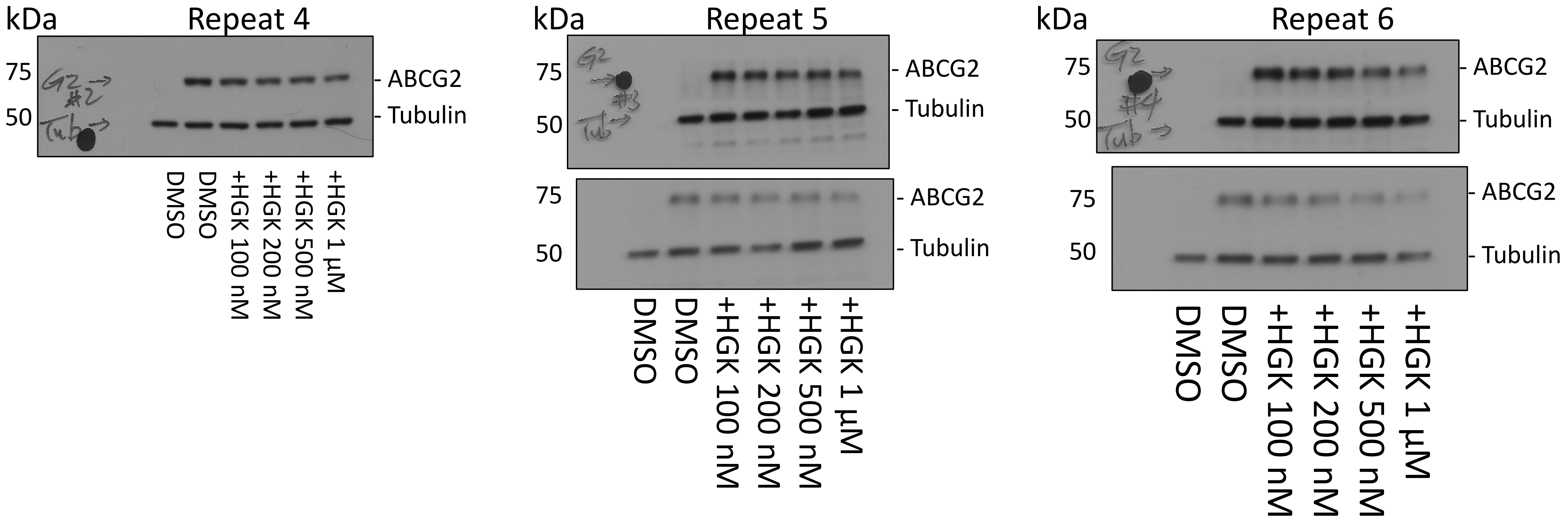

Original western blots for Figure 5A (NCI-H460/MX20)

Figure S1: Continued

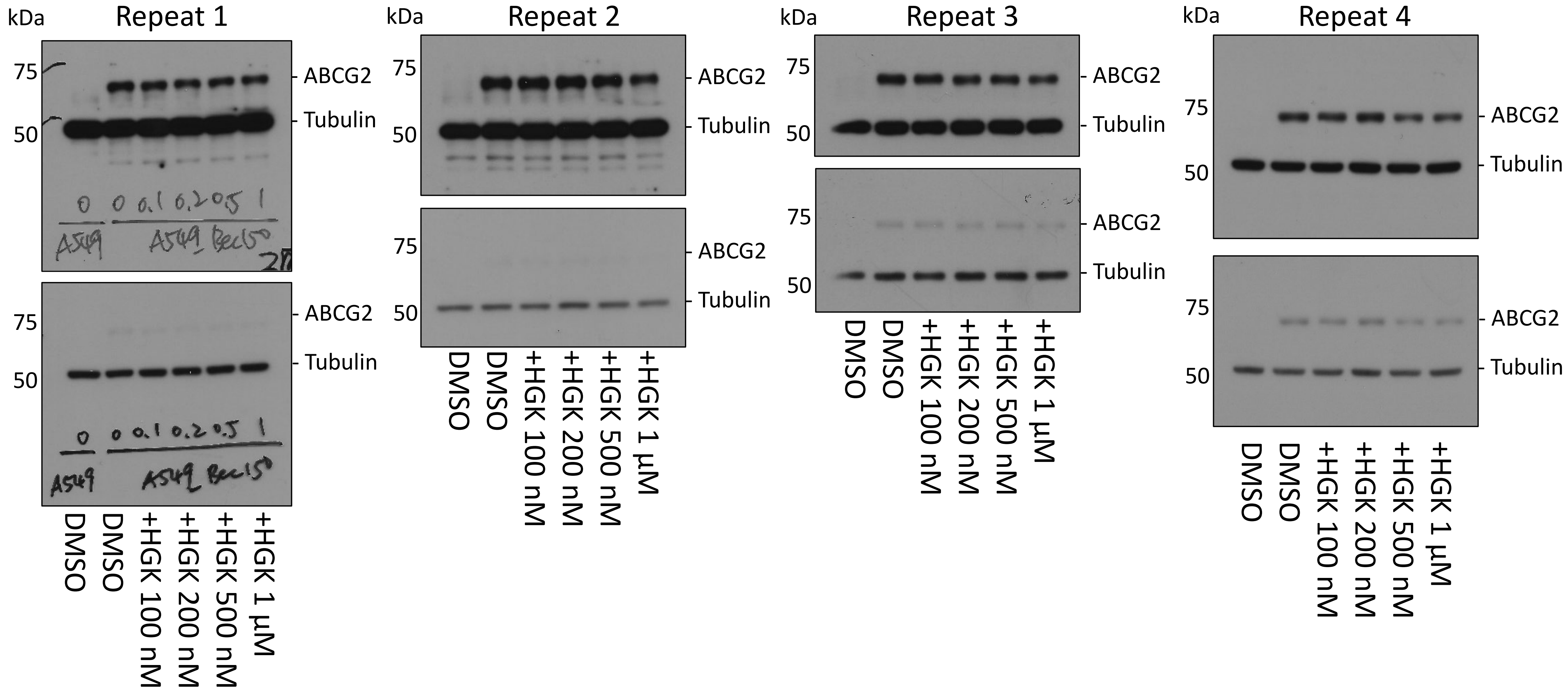

Original western blots for Figure 5B (A549-Bec150)

Figure S1: Continued

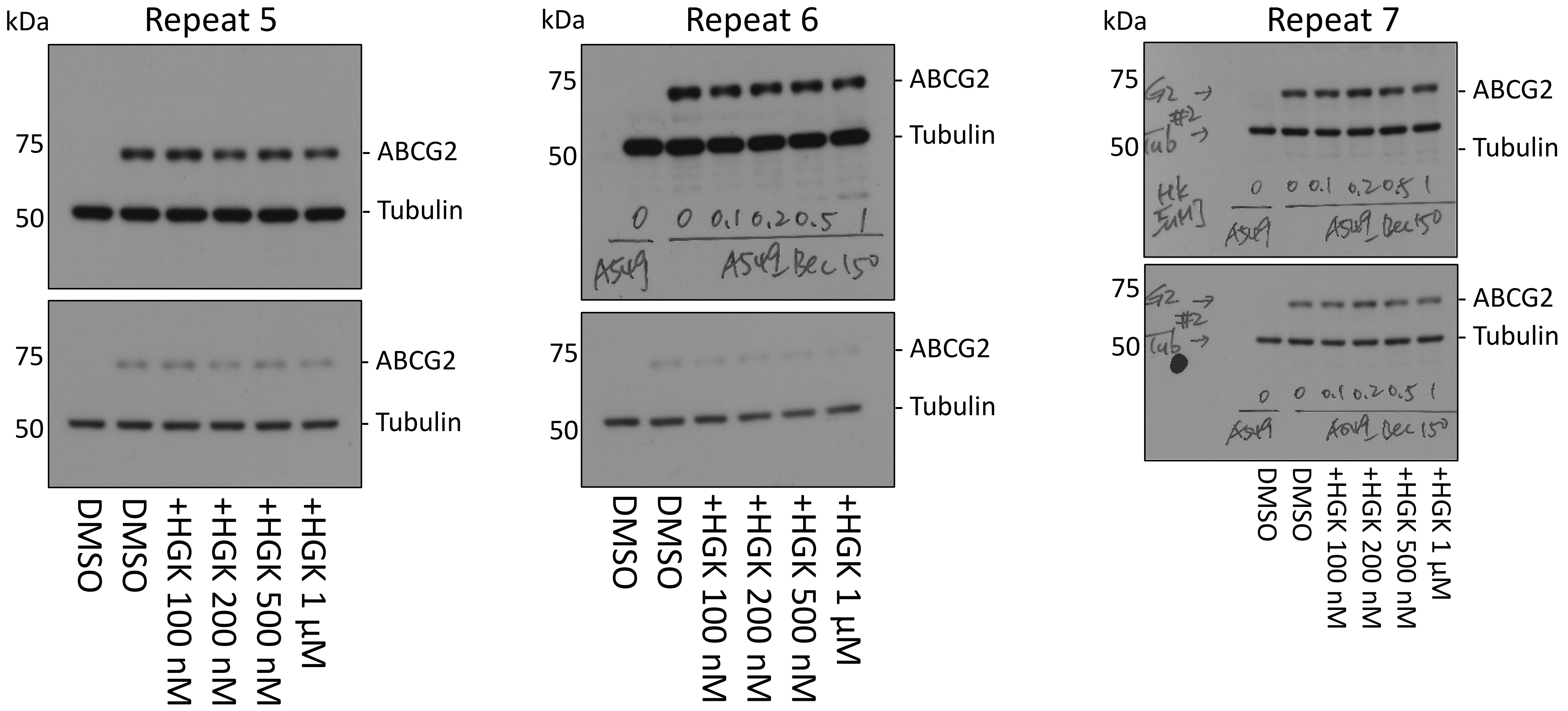

Original western blots for Figure 5B (A549-Bec150)
